# Estimating the causal effects of genetically predicted plasma proteome on heart failure

**DOI:** 10.3389/fcvm.2023.978918

**Published:** 2023-02-13

**Authors:** Jian Yang, Bin Yan, Haoxuan Zhang, Qun Lu, Lihong Yang, Ping Liu, Ling Bai

**Affiliations:** ^1^Clinical Research Center, The First Affiliated Hospital of Xi’an Jiaotong University, Xi'an, China; ^2^School of Life Science and Engineering, Southwest Jiaotong University, Chengdu, China; ^3^Department of Cardiology, The First Affiliated Hospital of Xi’an Jiaotong University, Xi'an, China

**Keywords:** heart failure, plasma proteome, Mendelian randomization, met, CD209, USP25

## Abstract

**Background:**

Heart Failure (HF) is the end-stage cardiovascular syndrome with poor prognosis. Proteomics holds great promise in the discovery of novel biomarkers and therapeutic targets for HF. The aim of this study is to investigate the causal effects of genetically predicted plasma proteome on HF using the Mendelian randomization (MR) approach.

**Methods:**

Summary-level data for the plasma proteome (3,301 healthy individuals) and HF (47,309 cases; 930,014 controls) were extracted from genome-wide association studies (GWASs) of European descent. MR associations were obtained using the inverse variance-weighted (IVW) method, sensitivity analyses, and multivariable MR analyses.

**Results:**

Using single-nucleotide polymorphisms as instrumental variables, 1-SD increase in MET level was associated with an approximately 10% decreased risk of HF (odds ratio [OR]: 0.92; 95% confidence interval [CI]: 0.89 to 0.95; *p* = 1.42 × 10^−6^), whereas increases in the levels of CD209 (OR: 1.04; 95% CI: 1.02–1.06; *p* = 6.67 × 10^−6^) and USP25 (OR: 1.06; 95% CI: 1.03–1.08; *p* = 7.83 × 10^−6^) were associated with an increased risk of HF. The causal associations were robust in sensitivity analyses, and no evidence of pleiotropy was observed.

**Conclusion:**

The study findings suggest that the hepatocyte growth factor/c-MET signaling pathway, dendritic cells-mediated immune processes, and ubiquitin-proteasome system pathway are involved in the pathogenesis of HF. Moreover, the identified proteins have potential to uncover novel therapies for cardiovascular diseases.

## 1. Introduction

Heart failure (HF) is a life-threatening clinical syndrome that represents the end stage of various cardiac conditions, including ischemic heart disease, hypertension, and non-ischemic cardiomyopathy (1). HF is a leading cause of cardiovascular hospitalization and death worldwide, especially in individuals older than 60 years (2, 3). The common risk factors of HF include hypertension, hypercholesterolaemia, diabetes, obesity, familial history of HF, and psychological agents (4–7). Despite remarkable advances in HF treatment, the prognosis of patients with HF remains poor, and none of the treatments has been proven to be effective for acute HF and HF with preserved ejection fraction (8, 9). Discovering novel biomarkers for early diagnosis or etiological treatment has always been a central goal for specialists in this field (10).

Current omics techniques, particularly proteomics, are holding a revolution in the search for clinically useful biomarkers for complex human diseases (11, 12). Proteins are macromolecules with biological functions in organisms and can also serve as intermediate phenotypes for how genetic and non-genetic factors act on diseases. The advent of proteomic technologies has allowed simultaneous quantification of thousands of proteins in human cells, blood, and tissues, in stark contrast to previous biomarker research that focused on single or several protein measurements (13). Proteomics has been increasingly applied to identify novel biomarkers, reveal pathophysiological mechanisms, and develop novel therapeutic targets for cardiovascular diseases since the late 1990s (14–17). Furthermore, improvements in proteomic techniques and integration with genomics have provided broader application prospects for proteomics.

Mendelian randomization (MR) is a genetic epidemiological study design that uses genetic variants as instrumental variables to investigate causal inferences between modifiable exposures and disease outcomes (18). The MR works analogous to a randomized controlled trial, except that the population is randomly assigned to different levels of exposure by genotypes (19). Given the fact that genotypes are determined at birth and, therefore, not susceptible to confounding and reverse causation, MR has the potential to provide an unbiased investigation of the causal effect of a modifiable exposure on a disease outcome of interest (20). Recently, genome-wide association studies (GWASs) have been introduced in the human plasma proteome and have evaluated the associations of single-nucleotide polymorphisms (SNPs) with thousands of proteins, which provides a great opportunity to investigate the causal inferences between the human plasma proteome and HF (21–23). The present study aimed to provide a comprehensive review of the causal effects of genetically predicted human plasma proteome (including 2,994 proteins) on HF by extracting summary-level data from large GWASs.

## 2. Methods

### 2.1. Study design

**Figure 1:**
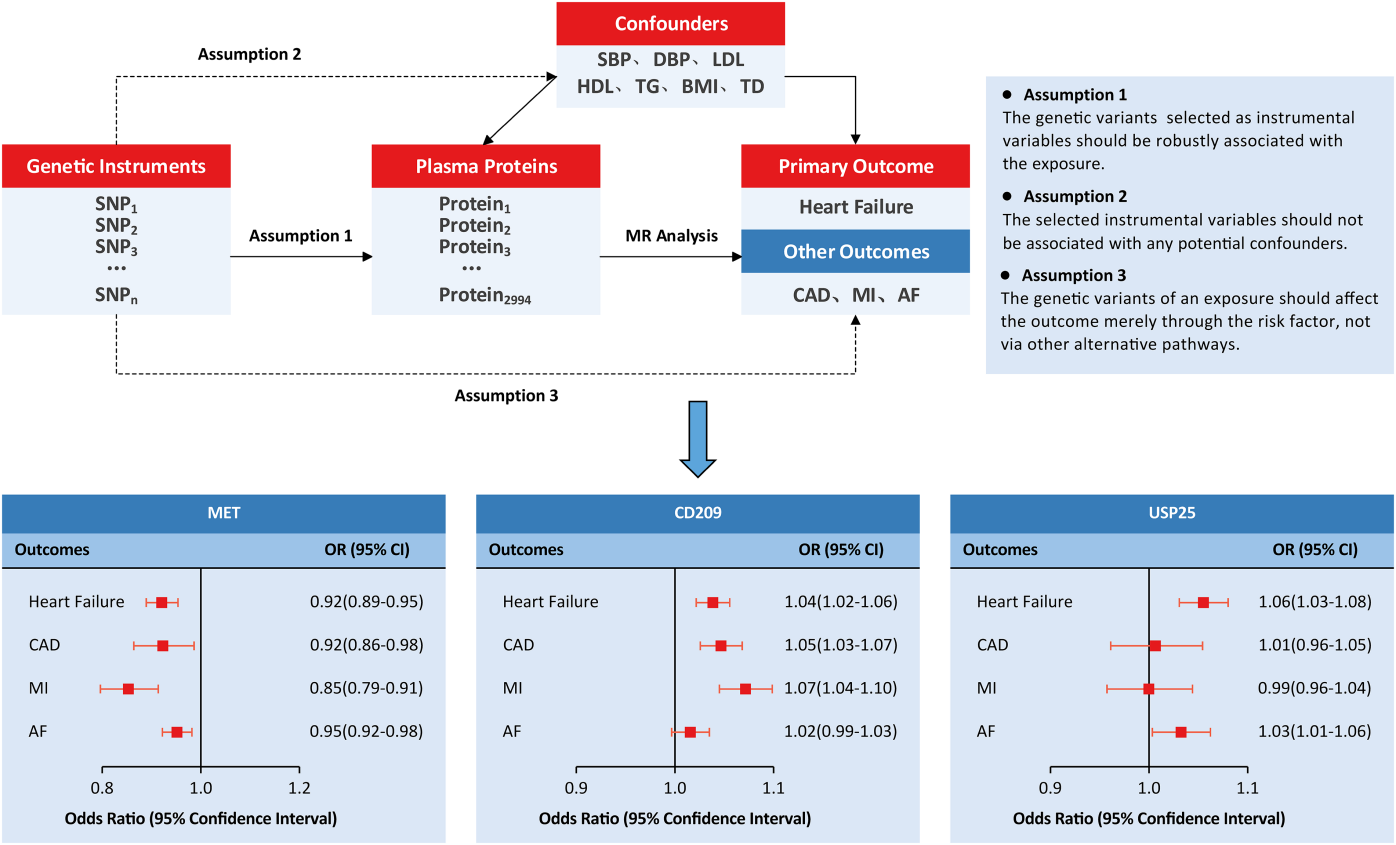
Study design and principal findings of the MR investigation. AF, atrial fibrillation; CAD, coronary artery disease; MI, myocardial infarction.

We employed an MR study design based on publicly available summary statistics from large-scale GWASs (Figure 1). In this study, genetically predicted human plasma proteomes were used as exposures; genetic associations with HF were selected as primary outcomes; and other outcomes included coronary artery disease (CAD), myocardial infarction (MI), and atrial fibrillation (AF). In addition, the causal associations were tested by adjusting for several specific confounders/mediums, including circulating lipid levels (low-density lipoprotein [LDL], high-density lipoprotein [HDL], triglycerides [TG]), blood pressure traits (systolic blood pressure [SBP] and diastolic blood pressure [DBP]), body mass index (BMI), and type 2 diabetes (T2D).

### 2.2. Ethical approval

Ethical approval and written informed consent were not sought because all datasets included in this study were extracted from publicly available GWASs.

### 2.3. Study population and data sources

Table 1 summarizes the data sources used in MR analysis. Genetic instruments for exposure were taken from a recent GWAS of the human plasma proteome (21). The study population comprised 3,301 healthy blood donations from 25 centers across England. Proteins were quantified using an aptamer-based SOMAscan assay. Log-transformed protein levels of 3,283 SOMAmers (mapping to 2,994 unique proteins) were tested by adjusting for age, sex, duration between blood draw and processing, and ancestry in GWAS analysis. GWAS summary statistics for HF were derived from the Heart Failure Molecular Epidemiology for Therapeutic Targets (HERMES) Consortium (24), comprising 47,309 cases and 930,014 controls of European ancestry. Cases were recruited according to definite clinical criteria without definition based on the left ventricular ejection fraction. Summary statistics for CAD (60,801 cases and 123,504 controls) and MI (60,801 cases and 123,504 controls) were obtained from the CARDIoGRAMplusC4D Consortium (25). Summary statistics for AF were obtained from a large GWAS on 65,446 cases and 522,744 controls, of which 84.2% were European (26). Genetic associations with LDL, HDL, and TG were obtained from the Global Lipids Genetics Consortium that included 188,578 European individuals (28). Genetic associations with SBP and DBP were obtained from the UK Biobank including 757,601 European individuals (27). Genetic summary-level data for BMI (694,649 individuals of European ancestry) were obtained from the Lindgren’s group in Oxford University (29), and genetic data for T2D (180,834 cases and 1,159,055 controls) were obtained from the Diabetes Meta-Analysis of Trans-Ethnic association studies (DIAMANTE) Consortium (30).

**Table 1:** Descriptive characteristics of the GWASs included in the Mendelian randomization analyses.

Variable	Phenotype	Sample Size	Ancestry	Study
Exposure	Plasma proteome (2,994 proteins)	3,301 individuals	European	(21)
Primary outcome	Heart failure	47,309 cases/930,014 controls	European	(24)
Other outcomes	Coronary artery disease	60,801 cases/123,504 controls	European	(25)
	Myocardial infarction	43,676 cases/123,504 controls	European	(25)
	Atrial fibrillation	65,446 cases/522,744 controls	Multi-ancestry	(26)
Confounders	SBP, DBP	757,601 individuals	European	(27)
	LDL, HDL, TG	188,578 individuals	European	(28)

SBP, systolic blood pressure; DBP, diastolic blood pressure; LDL, low-density lipoprotein; HDL, high-density lipoprotein; TG, triglycerides.

### 2.4. Statistical analysis

To obtain genetic instruments for the 2,994 plasma proteins, we extracted all SNPs that had reached a significance threshold of *p* < 1 × 10^−5^. Next, we performed a clumping procedure to select for independence, setting a linkage disequilibrium (LD) threshold of *r*^2^ < 0.001 in a 10-Mb window in the 1,000 Genomes Project Phase 3 (EUR) reference panel. Proxy SNPs (LD *r*^2^ > 0.8) were used when no instrument SNP for predicting protein level was available in the outcome dataset. The strength of each genetic instrument was evaluated using two key parameters: the proportion of variance explained by the SNPs (R^2^) and the F statistic.

The inverse variance-weighted (IVW) method was adopted for the primary MR analysis. The IVW method can be equivalently regarded as a weighted regression of SNP-outcome effects on SNP-exposure effects, with the intercept constrained to zero. However, the IVW estimate is known to suffer from horizontal pleiotropy bias, where any SNP acts on the outcome through pathways other than the exposure. Therefore, several additional MR methods were used to account for such bias, including the weighted median method, which allowed no more than 50% of the SNPs to be invalid instruments (31) and the Egger method, which could detect and adjust for pleiotropy by transforming the intercept to be non-zero (32). Furthermore, we removed horizontal pleiotropic outliers using the MR-PRESSO method and evaluated the presence of horizontal pleiotropy using the MR-Egger intercept test (33), Cochran Q test (34), and leave-one-out analyses (35).

Multivariable MR analysis was conducted to assess the potential confounding effect of circulating lipids (LDL, HDL, and TG) and blood pressure traits (SBP and DBP). Genetic instruments for each trait were extracted and combined with those for the proteins. The IVW method was employed to correct for confounders in the multivariable MR model. All analyses were performed using the TwoSampleMR and MVMR packages in R software (version 3.6.1; R Foundation for Statistical Computing, Vienna, Austria). Causal estimates are expressed as odds ratios (ORs) with 95% confidence intervals (CIs) per 1-SD increase in quantification of each protein on outcome. Statistical significance was set at a multiple-testing corrected threshold of *p* < 1.52 × 10^−5^ (0.05/3283) following the Bonferroni method.

## 3. Results

### 3.1. Main MR analysis

**Figure 2:**
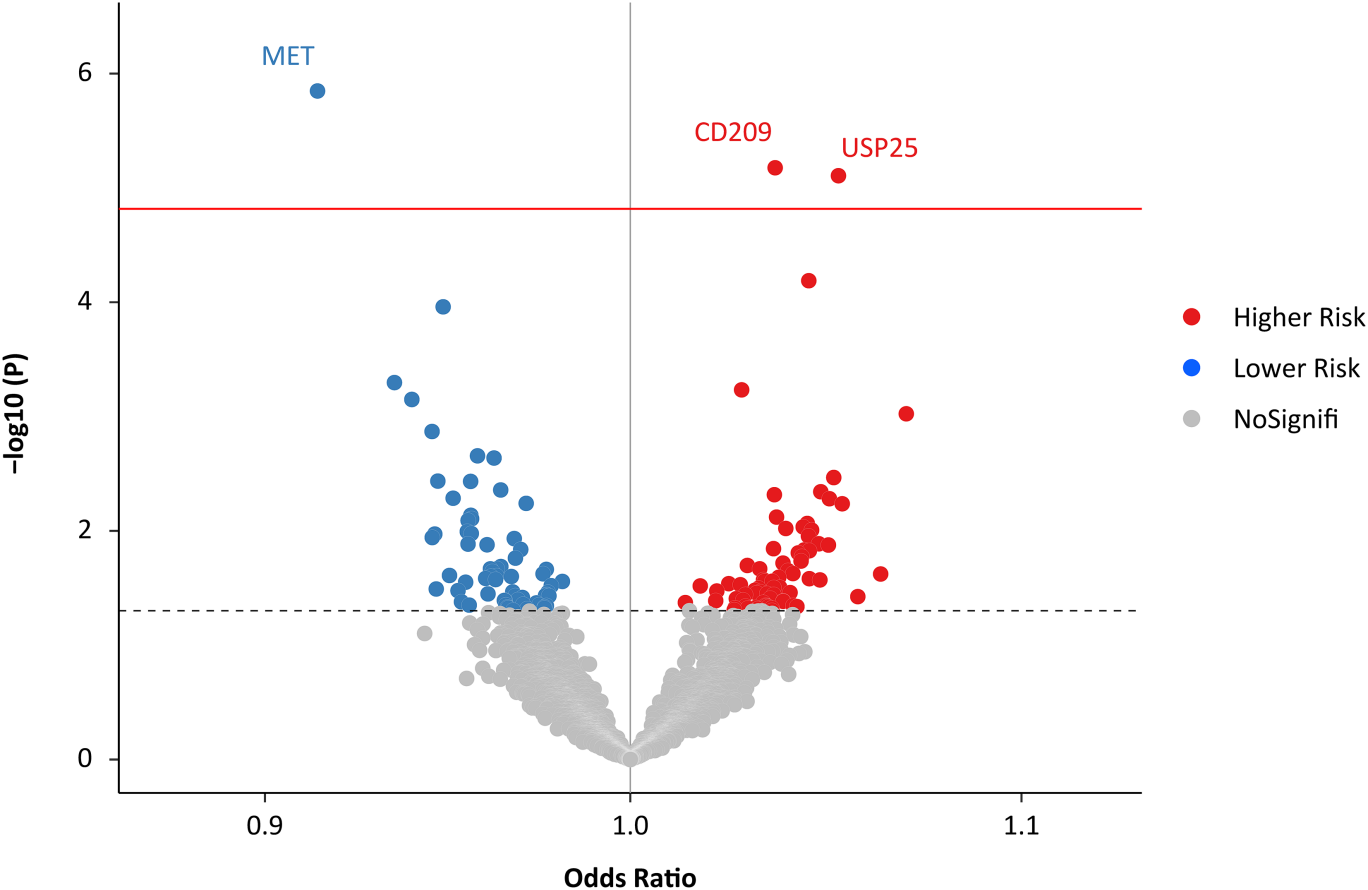
Effects of genetically predicted plasma proteome on HF. The red solid line represents the Bonferroni-corrected significant threshold of *p* = 1.52 × 10^−5^. The black dotted line represents the suggestive association threshold of *p* = 0.05. HF, heart failure.

**Figure 3:**
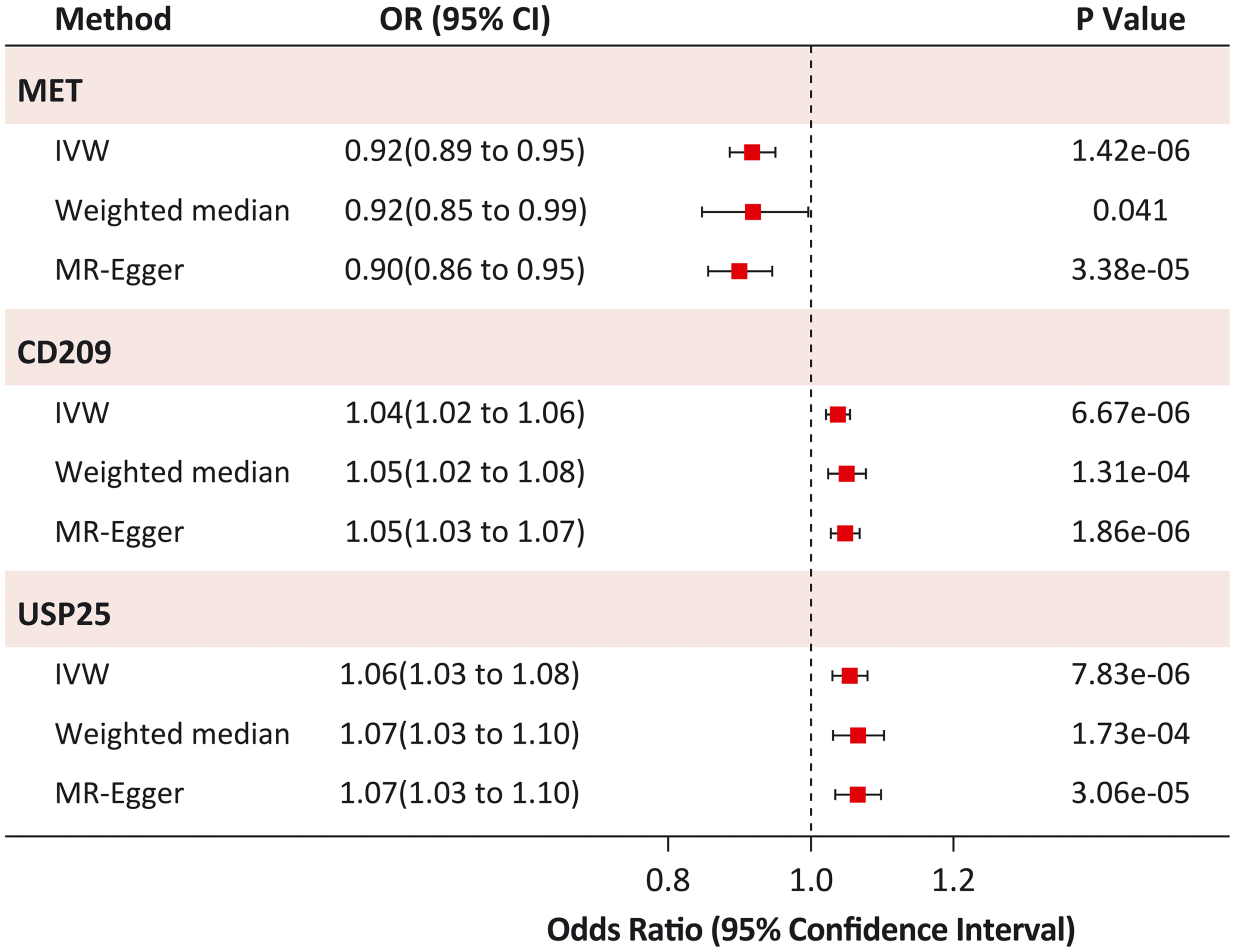
Sensitivity analysis of causal associations between identified proteins and Heart Failure. IVW, inverse variance-weighted.

The IVW MR analysis identified three proteins that were causally associated with HF (Figure 2; Supplementary Table S1). Using 14 SNPs as instrumental variables (variance explained = 12.5%; F statistic = 33.5; Supplementary Table S2), genetically predicted increased levels of MET were associated with approximately 10% decreased risk of HF (IVW OR: 1.16; 95% CI: 1.02 to 1.34; *p* = 1.42 × 10^−6^; Figure 3; Supplementary Figure S1). Increased levels of CD209 (IVW OR: 1.04; 95% CI: 1.02 to 1.06; *p* = 6.67 × 10^−6^) and USP25 (IVW OR: 1.06; 95% CI: 1.03–1.08; *p* = 7.83 × 10^−6^) were associated with increased risk of HF (Figure 3; Supplementary Figures S2, S3), with 22 SNPs explaining 48.0% variance (F statistic = 137.6) of CD209 and 17 SNPs explaining 24.0% variance (F statistic = 66.9) of USP25 (Supplementary Tables S3, S4).

### 3.2. Sensitivity analysis

The causal risk of MET on HF was robust in the sensitivity analysis (weighted median OR: 0.92; 95% CI: 0.85 to 0.99; *p* = 0.041; MR-Egger OR: 0.90; 95% CI: 0.86 to 0.95; *p* = 3.38 × 10^−5^; Figure 3). Horizontal pleiotropy was not observed in the MR-Egger intercept test (*p* = 0.973), Cochran Q test (Q statistic = 14.4; *p* = 0.349), or leave-one-out analyses (Supplementary Figure S4). Similar results were found for the causal risk of CD209 on HF (weighted median OR: 1.05; 95% CI: 1.02 to 1.08; *p* = 1.31 × 10^−4^; MR-Egger OR: 1.05; 95% CI: 1.03 to 1.07; *p* = 1.86 × 10^−6^), and USP25 on HF (weighted median OR: 1.07; 95% CI: 1.03 to 1.10; *p* = 1.73 × 10^−4^; MR-Egger OR: 1.07; 95% CI: 1.03 to 1.10; *p* = 3.06 × 10^−5^). The MR-Egger intercept test (*p* > 0.05) and Cochran’s Q test (*p* > 0.05) did not indicate any evidence of pleiotropy for the causal effects of CD209 and USP25 on HF. Leave-one-out analyses suggested that the effect of CD209 on HF was substantially driven by a single SNP rs505922 (Supplementary Figure S5), whereas the effect of USP25 on HF was robust (Supplementary Figure S6).

### 3.3. Multivariable MR analysis

To verify the direct causal effects of MET, CD209, and USP25 on HF, we performed multivariable MR analyses adjusting for common HF risk factors. The causal effects of MET on HF were broadly consistent after adjusting for LDL (OR: 0.93; 95% CI: 0.88 to 0.98, *p* = 9.99 × 10^−3^), HDL (OR: 0.89; 95% CI: 0.83 to 0.95, *p* = 1.21 × 10^−3^), TG (OR: 0.89; 95% CI: 0.83 to 0.95, *p* = 1.43 × 10^−3^), SBP (OR: 0.93; 95% CI: 0.89 to 0.98, *p* = 6.69 × 10^−3^), DBP (OR: 0.93; 95% CI: 0.90 to 0.96, *p* = 1.42 × 10^−4^), BMI (OR: 0.97; 95% CI: 0.93 to 0.99, *p* = 0.036), and T2D (OR: 0.94; 95% CI: 0.91 to 0.98, *p* = 0.002; Table 2). Similar results were observed for the effect of CD209 on HF (OR: 1.04, 95% CI: 1.01 to 1.06 and *p* = 5.77 × 10^−3^ for LDL; OR: 1.05, 95% CI: 1.02 to 1.08 and *p* = 4.45 × 10^−3^ for HDL; OR: 1.05, 95% CI: 1.02 to 1.08 and *p* = 5.43 × 10^−3^ for TG; OR: 1.04, 95% CI: 1.02 to 1.06 and *p* = 6.67 × 10^−6^ for SBP; OR: 1.04, 95% CI: 1.02 to 1.07 and *p* = 9.86 × 10^−4^ for DBP; OR: 1.02, 95% CI: 1.01 to 1.04 and *p* = 0.029 for BMI; OR: 1.03, 95% CI: 1.01 to 1.05 and *p* = 0.011 for TD; Table 2). However, the causal effects of USP25 on HF (OR: 1.02, 95% CI: 0.97 to 1.08 and *p* = 0.393 for LDL; OR: 1.04, 95% CI: 0.97 to 1.12 and *p* = 0.250 for HDL; OR: 1.04, 95% CI: 0.97 to 1.12 and *p* = 0.296 for TG; OR: 1.06, 95% CI: 1.03 to 1.08 and *p* = 7.83 × 10^−6^ for SBP; OR: 1.04, 95% CI: 1.01 to 1.08 and *p* = 0.016 for DBP; OR: 0.99, 95% CI: 0.96 to 1.04 and *p* = 0.793 for BMI; OR: 1.04, 95% CI: 1.01 to 1.07 and *p* = 0.017 for TD; Table 2) became non-significant after adjusting for LDL, HDL, TG, or BMI suggesting that circulating lipid traits and BMI might have a confounding effect on the causal association between USP25 and HF.

**Table 2:** Multivariable Mendelian randomization associations of risk proteins with heart failure adjusting for potential confounders.

Model	MET		CD209		USP25	
	OR(95% CI)	*P*	OR(95% CI)	*P*	OR(95% CI)	*P*
Unadjusted model	0.92 (0.89,0.95)	1.42e-06	1.04 (1.02,1.06)	6.67e-06	1.06 (1.03,1.08)	7.83e-06
Adjusted for SBP	0.93 (0.89,0.98)	6.69e-03	1.04 (1.02,1.07)	9.86e-04	1.04 (1.01,1.08)	0.016
Adjusted for DBP	0.93 (0.90,0.96)	1.42e-04	1.04 (1.02,1.06)	5.02e-05	1.04 (1.02,1.07)	1.05e-03
Adjusted for LDL	0.93 (0.88,0.98)	9.99e-03	1.04 (1.01,1.06)	5.77e-03	1.02 (0.97,1.08)	0.393
Adjusted for HDL	0.89 (0.83,0.95)	1.21e-03	1.05 (1.02,1.08)	4.45e-03	1.04 (0.97,1.12)	0.250
Adjusted for TG	0.89 (0.83,0.95)	1.43e-03	1.05 (1.02,1.08)	5.43e-03	1.04 (0.97,1.12)	0.296
Adjusted for BMI	0.97 (0.93,0.99)	0.036	1.02 (1.01,1.04)	0.029	0.99 (0.96,1.04)	0.793
Adjusted for T2D	0.94 (0.91,0.98)	0.002	1.03 (1.01,1.05)	0.011	1.04 (1.01,1.07)	0.017

SBP, systolic blood pressure; DBP, diastolic blood pressure; LDL, low-density lipoprotein; HDL, high-density lipoprotein; TG, triglycerides.

### 3.4. Associations with other outcomes

We further investigated the causal effects of MET, CD209, and USP25 on the three relevant outcomes. Genetically predicted MET levels showed consistent associations with CAD (OR: 0.92; 95% CI: 0.86 to 0.98), MI (OR: 0.85; 95% CI: 0.79 to 0.91), and AF (OR: 0.95; 95% CI: 0.92 to 0.98; Figure 1). Genetically predicted levels of CD209 were associated with CAD (OR: 1.05; 95% CI: 1.03–1.07) and MI (OR: 1.07; 95% CI: 1.04 1.10). Genetically predicted levels of USP25 were associated with AF (OR: 1.03; 95% CI: 1.01 to 1.06).

## 4. Discussion

In this comprehensive MR analysis of the effect of the human plasma proteome on HF, we identified three plasma proteins that might have causal associations with HF. Genetically predicted higher level of MET was associated with a decreased risk of HF, whereas higher levels of CD209 and USP25 were associated with an increased risk of HF. The results were robust in alternative MR methods and sensitivity analyses. Multivariable MR analyses showed the effects of MET and CD209 on HF were robust after adjustment for confounding factors, whereas lipid traits (LDL, HDL, and TG) might have a confounding effect on the association between USP25 and HF. Associations with other cardiovascular outcomes suggested that MET might also have causal effects on CAD, MI, and AF, CD209 might have effects on CAD and MI, USP25 might have a causal effect on AF.

Several published studies have investigated the association between high-throughput proteomics and HF risk based on prospective cohorts (36–38). However, the approach in our study is significantly different from these previous approaches. First, we implemented an MR study design that made causal inferences from the perspective of genetics. Unlike previous observational studies, the MR study design was able to provide etiological clues for revealing the underlying pathogenesis of HF and was less susceptible to confounding factors, such as dietary habits, medications, and comorbidities. Second, we extracted data from the largest GWAS for HF. With a large sample size (47,309 cases and 930,014 controls) and wide population coverage, the findings of our study are highly powerful and generalizable. In addition, the plasma proteome included in our analysis covered a wide range of approximately 3,000 proteins using the latest proteomic profiling platform with high sample throughput and sensitivity of detection. Third, the proteome could serve as an intermediate phenotype of the genetic risk factors and disease outcome, which might help to uncover the underlying molecular pathways that connect the genome to HF.

Our study reported three proteins (MET, CD209, and USP25) that might have causal effects on HF. Interestingly, MET has long been suggested to play a role in cardiovascular disease in previous studies (39, 40). MET, also known as c-MET, is a hepatocyte growth factor (HGF) receptor. The HGF/c-MET function plays a prominent role in protecting the heart from both acute and chronic insults, including ischemic injury and doxorubicin-induced cardiotoxicity (39). This mechanism may be involved in enhancing the ability of cardiac stem cells (41), attenuating cardiac hypertrophy, remodeling (42), anti-calcification (43), anti-fibrotic (44), and anti-inflammatory (45). Consistent with these findings, our results showed that increased levels of MET had a beneficial effect on HF as well as on several other cardiovascular outcomes, thus providing novel clues for uncovering the pathogenesis or drug targets for cardiovascular diseases.

CD209 is a pathogen-recognition receptor expressed on the surface of immature dendritic cells (DCs) and is involved in the initiation of the primary immune response. A previous study found significant increases in the level of immature DCs (with CD209 as a marker) in the course of plaque progression in patients with atherosclerosis, especially in those with unstable atherosclerotic lesions (46). Another study showed that the immature type (CD 209 expression) of DCs was extensively recruited in the ischemic myocardium of patients after acute MI (47). Furthermore, DCs have been suggested to initiate an immune response against cardiac antigens in the infarcted myocardium, leading to progressive HF (47).

USP25 is a ubiquitin-specific protease, which represents the largest subfamily of deubiquitinating enzymes and plays essential roles in regulating the ubiquitin-proteasome system (UPS) (48). Actually, previous studies have suggested that the small ubiquitin-related modifier (SUMO) of SERCA2a, a critical ATPase responsible for Ca2+ re-uptake during excitation-contraction coupling, played an essential role in the development of HF (49, 50). Thus the UPS has the potential to serve as a novel target for future heart failure therapeutics (51–53).

Strengths of the study include the MR study design using data from large GWASs, use of comprehensive genomic atlas of the human plasma proteome, validation with multiple sensitivity analysis methods, and evaluation in other cardiovascular outcomes. This study also has several limitations. First, the data of plasma proteome are quantified using an aptamer-based SOMAscan assay. Though the aptamer-based strategy provides a rapid and convenient way of outsourcing protein measurements, some issues can still affect its accuracy, such as altered binding properties by electrical charge changes, protein structure alteration, and batch or plate effects. Second, the exposure-related instrumental variables are selected at a relatively relaxed threshold (*p* < 1 × 10^−5^), rather than the genome-wide significant threshold (*p* < 5 × 10^−8^), since the sample size of GWAS on proteome was not that large and few genome-wide significant SNPs were available for most proteins. Nevertheless, we evaluated the strength of these selected instrumental variables with the variance explained (R^2^) and the F statistic, and all instrumental variables were effective for declaring causal inferences. Third, the pathogenesis and therapies were much different for HF patients with reduced or preserved left ventricular ejection fraction. However, our study did not able to determine the causal roles of the three proteins on the two HF subtypes. Fourth, although our study identified novel biomarkers that might help to uncover novel drug targets or pathogenesis for HF, further studies were needed to verify the findings and the underlying mechanisms. Finally, the study samples involved in the MR analysis were restricted to European ancestry, further work should be done to verify these findings in other ethnic populations.

## 5. Conclusion

This MR investigation of causal associations between genetically predicted plasma proteome and HF found three proteins with causal effects on HF. Increased levels of MET appear to be associated with a lower risk of HF, whereas CD209 and USP25 may be associated with a higher risk of HF. The underlying mechanisms may be involved in the HGF/c-MET signaling pathway, DCs-mediated immune processes, and the UPS pathway. This study provides novel clues for uncovering the pathogenesis or drug targets in HF.

## Data availability statement

The original contributions presented in the study are included in the article/Supplementary material, further inquiries can be directed to the corresponding author.

## Ethics statement

Ethical review and approval were not required for the study on human participants in accordance with the local legislation and institutional requirements. Written informed consent for participation was not required for this study in accordance with the national legislation and the institutional requirements.

## Author contributions

JY and LB conceptualized and designed the study. JY and BY carried out the initial analyses and drafted the manuscript. HZ helped with the methodology. QL and LY contributed to the interpretation of results. PL and LB critically reviewed and revised the manuscript. All authors contributed to the article and approved the submitted version.

## Funding

The study was funded by General Projects of Social Development in Shaanxi Province (No. 2018SF-247).

## Conflict of interest

The authors declare that the research was conducted in the absence of any commercial or financial relationships that could be construed as a potential conflict of interest.

## Publisher’s note

All claims expressed in this article are solely those of the authors and do not necessarily represent those of their affiliated organizations, or those of the publisher, the editors and the reviewers. Any product that may be evaluated in this article, or claim that may be made by its manufacturer, is not guaranteed or endorsed by the publisher.
