# Supplementary material for: Estimating the causal effects of genetically predicted plasma proteome on heart failure
Source: Front Cardiovasc Med. 2023 Feb 13;10:978918. doi: 10.3389/fcvm.2023.978918 (PMC9968807; doi:10.3389/fcvm.2023.978918)
Supplement: Supplementary file 2 [file Data_Sheet_1.docx]

Supplementary Material

**Table S2. Genetic Instruments for MET and Associations with Heart Failure**

| **SNP ID** | **Explained R^2^ for MET** | **Explained R^2^ for HF** | **Effect allele (alternative)** | **β (Standard Error) for MET** | **β (Standard Error) for HF** |
| --- | --- | --- | --- | --- | --- |
| rs174530 | 0.007 | 5.67e-06 | A(G) | -0.128(0.026) | 0.019(0.008) |
| rs202236609 | 0.006 | 3.93e-07 | G(GT) | 0.428(0.096) | -0.029(0.046) |
| rs117837184 | 0.006 | 5.56e-08 | A(G) | 0.409(0.089) | -0.008(0.033) |
| rs78728395 | 0.006 | 1.07e-06 | T(G) | -0.422(0.095) | 0.034(0.033) |
| rs35990135 | 0.006 | 4.88e-07 | T(C) | -0.233(0.051) | 0.013(0.018) |
| rs138706986 | 0.006 | 5.49e-07 | T(C) | 0.416(0.093) | -0.024(0.033) |
| rs190126563 | 0.006 | 3.45e-08 | T(C) | 0.500(0.111) | -0.008(0.043) |
| rs12133766 | 0.006 | 1.58e-08 | A(G) | 0.242(0.054) | 0.002(0.016) |
| rs115719430 | 0.006 | 5.17e-09 | T(C) | -0.437(0.098) | -0.003(0.038) |
| rs72911482 | 0.006 | 2.13e-07 | T(C) | 0.360(0.080) | 0.014(0.031) |
| rs17864536 | 0.006 | 2.62e-06 | T(C) | 0.193(0.042) | -0.023(0.014) |
| rs4444509 | 0.007 | 6.81e-07 | A(G) | 0.135(0.028) | -0.007(0.009) |
| rs34999035 | 0.007 | 4.55e-08 | G(GT) | 0.192(0.040) | 0.004(0.020) |
| rs635634 | 0.042 | 2.91e-05 | T(C) | -0.380(0.032) | 0.056(0.010) |

HF = heart failure.

*Note.* All SNPs with P < 1×10^-5^ clumped at r^2^ < .001 with 10Mb.

**Table S3. Genetic Instruments for CD209 and Associations with Heart Failure**

| **SNP ID** | **Explained R^2^ for CD209** | **Explained R^2^ for HF** | **Effect allele (alternative)** | **β (Standard Error) for MET** | **β (Standard Error) for HF** |
| --- | --- | --- | --- | --- | --- |
| rs10887653 | 0.007 | 1.00e-06 | A(G) | 0.154(0.032) | 0.010(0.010) |
| rs111506384 | 0.007 | 3.69e-10 | T(C) | 0.442(0.094) | -0.001(0.032) |
| rs903212 | 0.006 | 2.53e-08 | A(G) | -0.166(0.036) | -0.002(0.014) |
| rs147241839 | 0.006 | 1.54e-06 | A(C) | -0.397(0.090) | 0.045(0.036) |
| rs8037496 | 0.007 | 0.000 | A(G) | -0.126(0.027) | 0.000(0.009) |
| rs151309093 | 0.006 | 4.04e-06 | T(C) | -0.379(0.086) | 0.059(0.03) |
| rs117901581 | 0.006 | 1.41e-06 | T(G) | 0.276(0.06) | 0.019(0.017) |
| rs17774369 | 0.006 | 8.50e-08 | T(C) | 0.284(0.064) | 0.007(0.023) |
| rs28594579 | 0.006 | 1.44e-07 | T(C) | -0.217(0.048) | -0.006(0.015) |
| rs17703342 | 0.006 | 1.13e-06 | A(G) | 0.204(0.046) | 0.015(0.014) |
| rs175093 | 0.006 | 1.45e-06 | A(G) | 0.138(0.030) | 0.012(0.010) |
| rs145827860 | 0.055 | 9.46e-08 | T(TTCTGGGGGC) | 0.415(0.030) | 0.005(0.015) |
| rs7248772 | 0.008 | 2.30e-06 | A(G) | -0.137(0.027) | 0.014(0.009) |
| rs10198199 | 0.006 | 3.28e-09 | A(G) | 0.147(0.033) | -0.001(0.011) |
| rs72847832 | 0.006 | 3.58e-08 | T(C) | -0.391(0.087) | -0.005(0.028) |
| rs77332351 | 0.006 | 4.77e-07 | A(G) | -0.248(0.056) | -0.011(0.017) |
| rs62425231 | 0.007 | 3.04e-06 | A(G) | -0.354(0.075) | -0.042(0.025) |
| rs12705056 | 0.008 | 1.27e-06 | T(C) | -0.172(0.034) | -0.014(0.012) |
| rs113640344 | 0.006 | 2.23e-07 | T(C) | -0.393(0.087) | -0.013(0.029) |
| rs505922 | 0.294 | 2.22e-05 | T(C) | -0.831(0.022) | -0.04(0.009) |
| rs3124759 | 0.009 | 2.32e-07 | A(G) | 0.184(0.033) | -0.005(0.011) |
| rs13300181 | 0.006 | 5.13e-07 | A(G) | 0.120(0.027) | 0.006(0.009) |

HF = heart failure.

*Note.* All SNPs with P < 1×10^-5^ clumped at r^2^ < .001 with 10Mb.

**Table S4. Genetic Instruments for USP25 and Associations with Heart Failure**

| **SNP ID** | **Explained R^2^ for USP25** | **Explained R^2^ for HF** | **Effect allele (alternative)** | **β (Standard Error) for MET** | **β (Standard Error) for HF** |
| --- | --- | --- | --- | --- | --- |
| rs11212330 | 0.006 | 8.01e-11 | T(C) | -0.310(0.068) | 0.001(0.023) |
| rs3918336 | 0.006 | 1.44e-06 | T(G) | -0.114(0.025) | -0.010(0.008) |
| rs6490710 | 0.007 | 1.51e-06 | A(G) | 0.181(0.038) | -0.014(0.012) |
| rs9576895 | 0.007 | 6.30e-07 | T(G) | 0.116(0.025) | -0.006(0.008) |
| rs3046018 | 0.007 | 6.75e-07 | CAA(C) | -0.133(0.028) | -0.011(0.013) |
| rs10683940 | 0.007 | 1.78e-08 | CAT(C) | -0.117(0.025) | -0.002(0.013) |
| rs2036736 | 0.006 | 9.14e-08 | A(C) | -0.126(0.027) | -0.003(0.009) |
| rs4632248 | 0.006 | 1.20e-06 | T(G) | -0.135(0.030) | -0.011(0.010) |
| rs6022640 | 0.006 | 2.61e-08 | T(C) | -0.129(0.029) | 0.002(0.009) |
| rs2235356 | 0.006 | 2.81e-07 | A(G) | 0.114(0.025) | 0.004(0.008) |
| rs6737649 | 0.006 | 7.13e-07 | A(G) | 0.180(0.040) | 0.011(0.013) |
| rs75331444 | 0.006 | 7.55e-06 | A(G) | -0.220(0.049) | -0.044(0.016) |
| rs4916495 | 0.008 | 1.92e-06 | T(C) | -0.158(0.031) | -0.014(0.010) |
| rs41316748 | 0.136 | 1.72e-05 | T(C) | -1.306(0.057) | -0.086(0.021) |
| rs117571913 | 0.007 | 3.48e-08 | A(G) | 0.472(0.096) | -0.006(0.032) |
| rs117203826 | 0.006 | 1.91e-07 | A(G) | -0.566(0.124) | -0.015(0.034) |
| rs11141531 | 0.006 | 3.40e-06 | A(C) | 0.155(0.033) | 0.021(0.011) |

HF = heart failure.

*Note.* All SNPs with P < 1×10^-5^ clumped at r^2^ < .001 with 10Mb.

**Figure S1. Scatterplot for Genetic Effect of MET on Heart Failure**

**
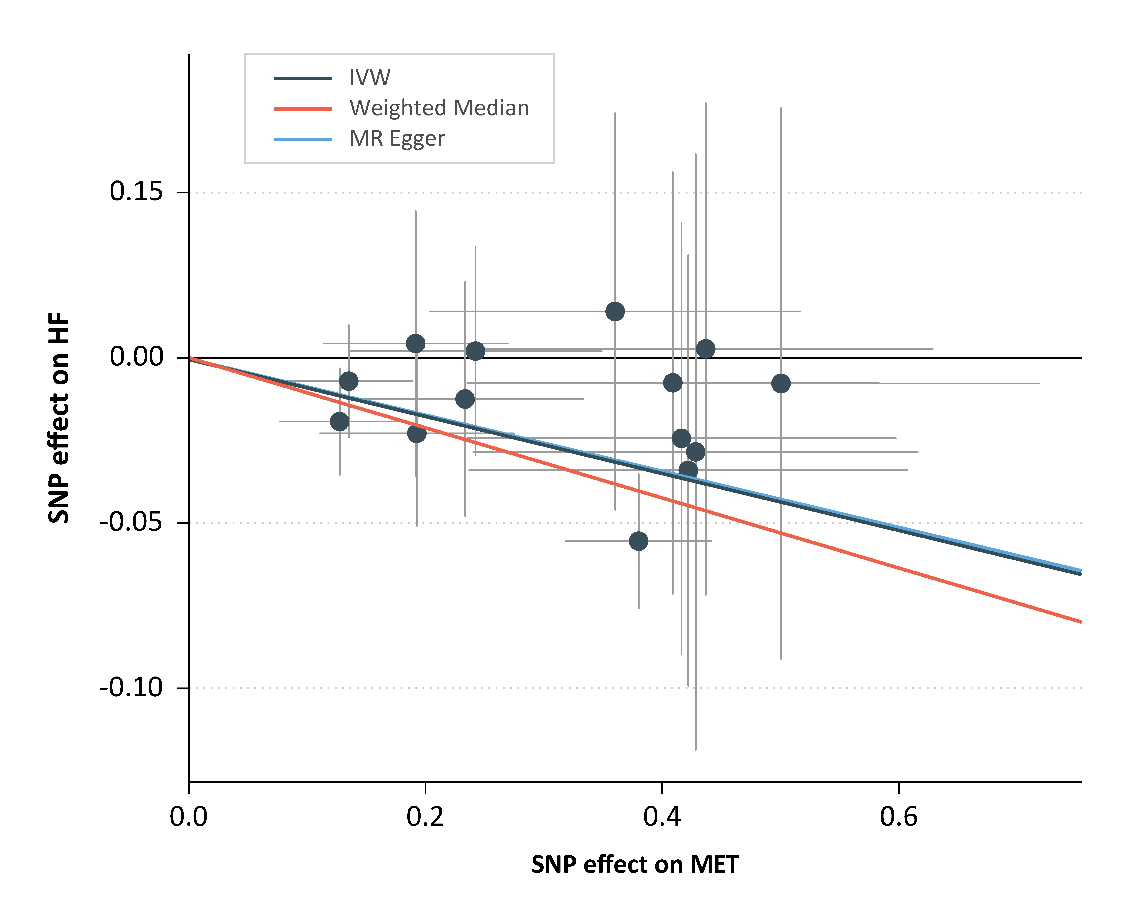
**

Scatterplot shows the MR-derived associations between genetically predicted level of MET with heart failure. Associations are calculated using the inverse variance–weighted (IVW), the weighted median and the MR-Egger methods. The slopes of each line represent the causal association for each method.

**Figure S2. Scatterplot for Genetic Effect of CD209 on Heart Failure**

**
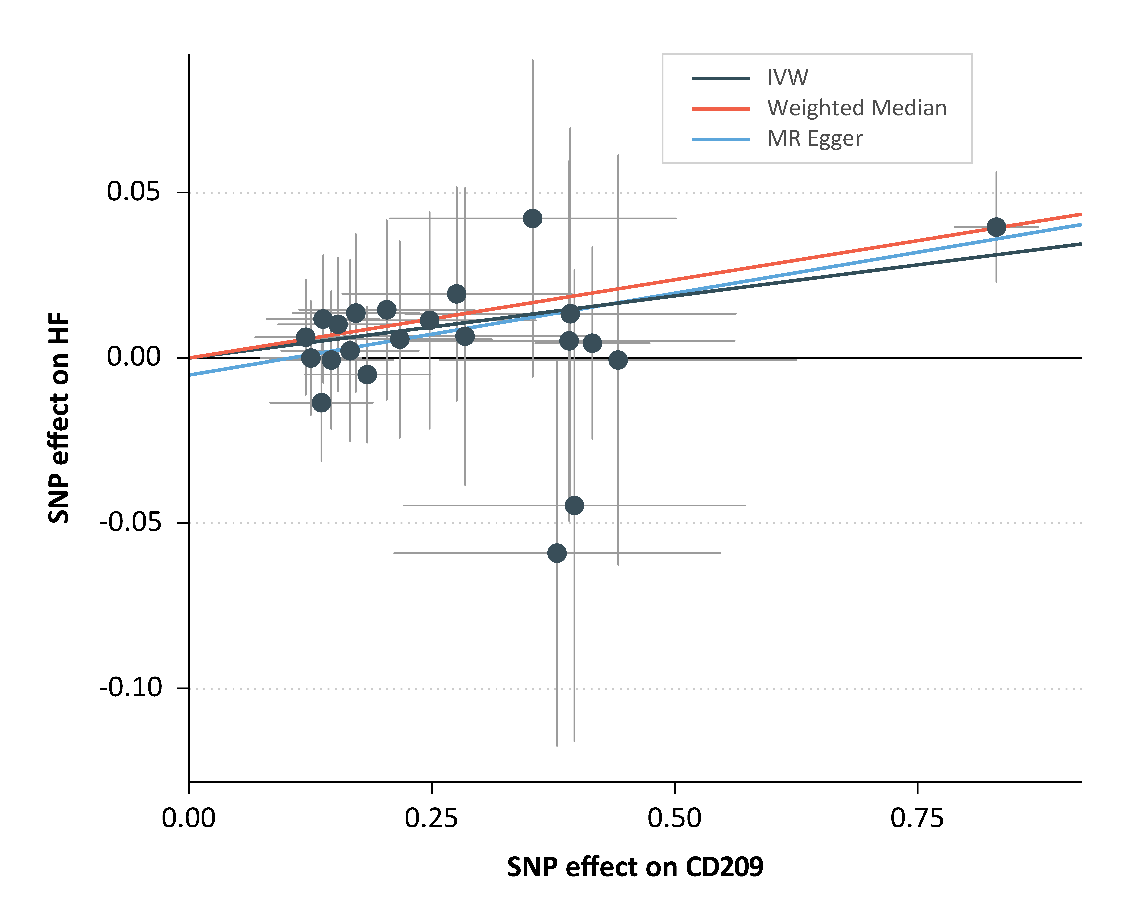
**

Scatterplot shows the MR-derived associations between genetically predicted level of CD209 with heart failure. Associations are calculated using the inverse variance–weighted (IVW), the weighted median and the MR-Egger methods. The slopes of each line represent the causal association for each method.

**Figure S3. Scatterplot for Genetic Effect of USP25 on Heart Failure**

**
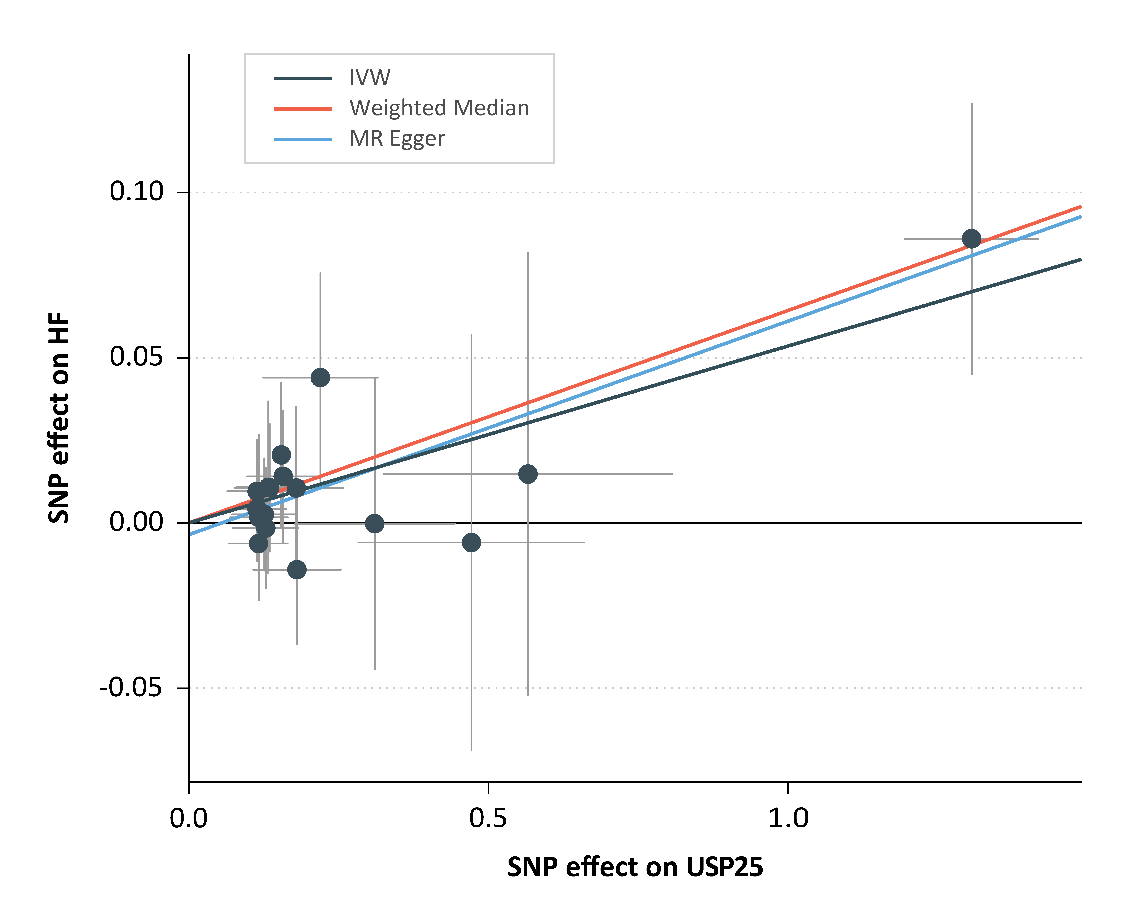
**

Scatterplot shows the MR-derived associations between genetically predicted level of USP25 with heart failure. Associations are calculated using the inverse variance–weighted (IVW), the weighted median and the MR-Egger methods. The slopes of each line represent the causal association for each method.

**Supplementary Figure 4. Leave-one-out Analysis for Causal Effect of MET on Heart Failure**

**
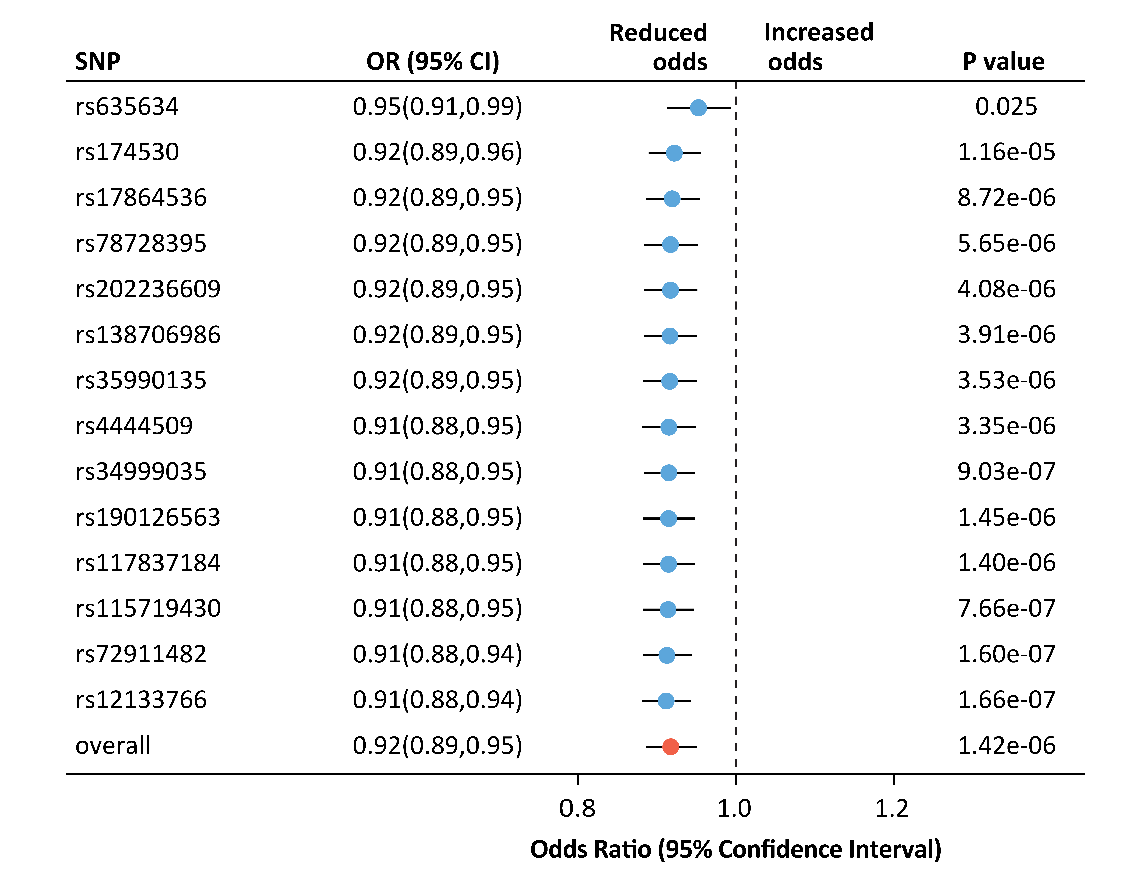
**

Leave-one-out analysis show the fluctuant MR associations of MET and heart failure by excluding each genetic instrument in turns.

**Supplementary Figure 5. Leave-one-out Analysis for Causal Effect of CD209 on Heart Failure**

**
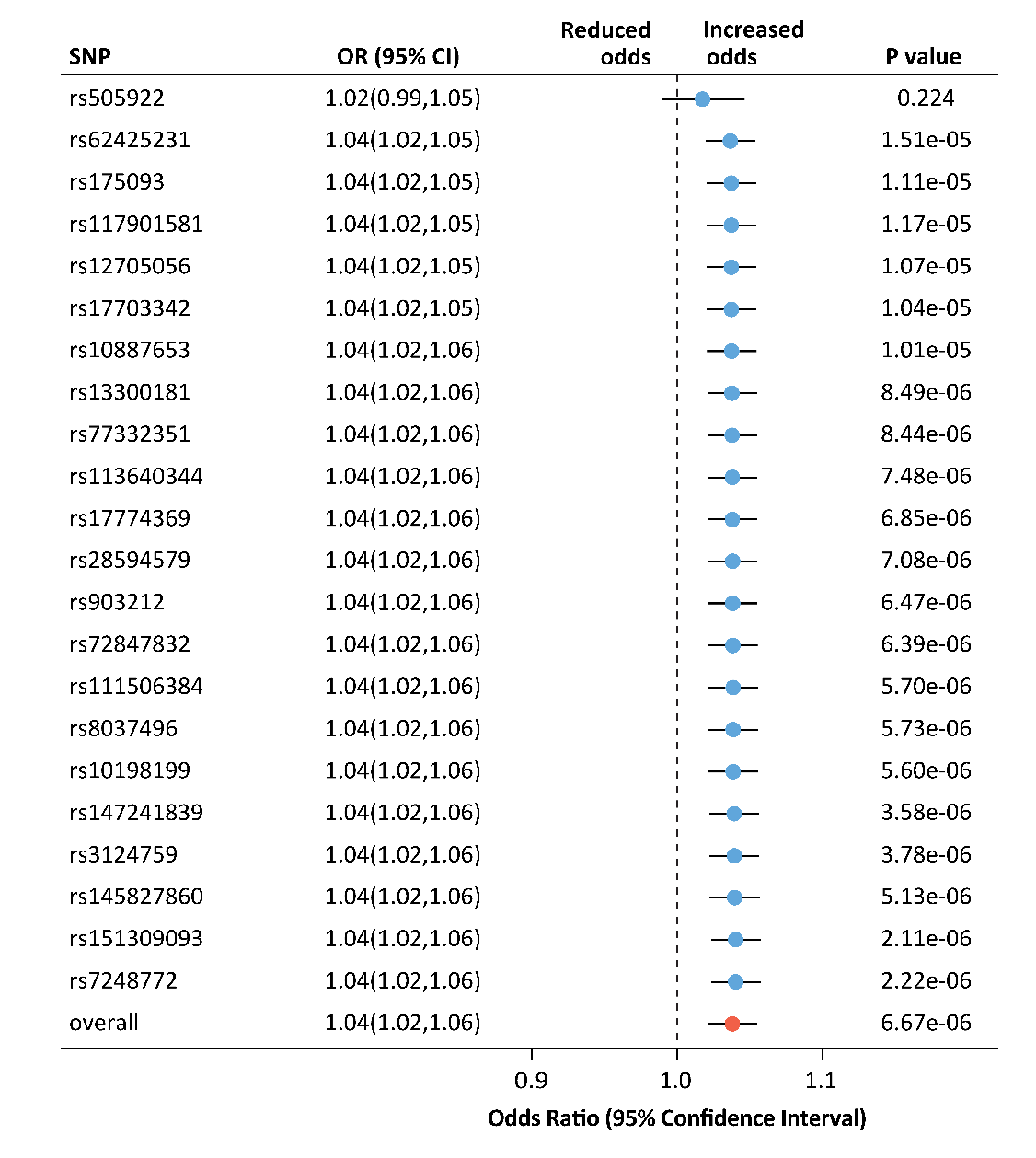
**

Leave-one-out analysis show the fluctuant MR associations of CD209 and heart failure by excluding each genetic instrument in turns.

**Supplementary Figure 6. Leave-one-out Analysis for Causal Effect of USP25 on Heart Failure**

**
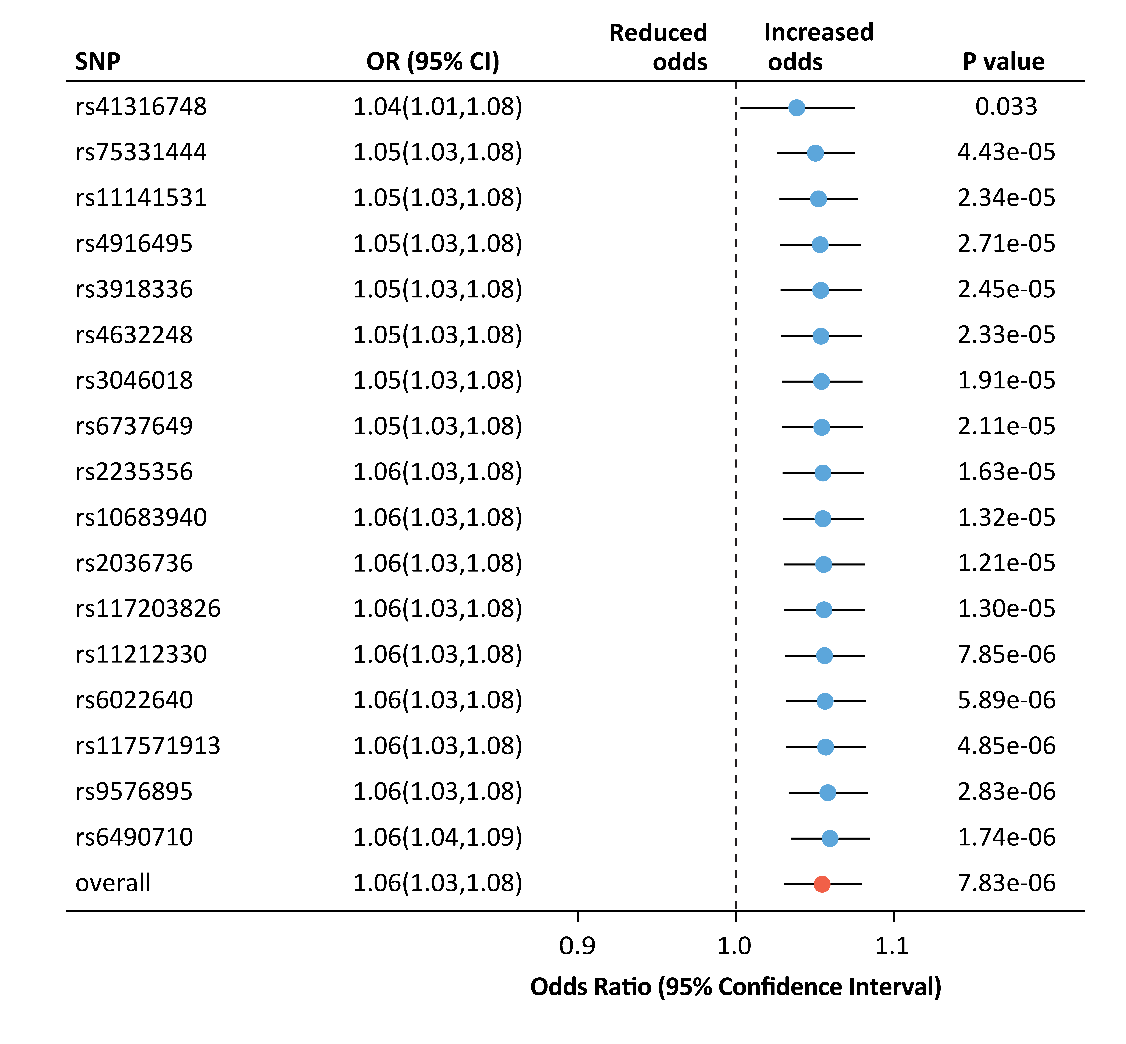
**

Leave-one-out analysis show the fluctuant MR associations of USP25 and heart failure by excluding each genetic instrument in turns.
